# Music is a distinct perceptual category with subjective grounds

**DOI:** 10.1038/s41598-026-54414-2

**Published:** 2026-05-27

**Authors:** Pauline Larrouy-Maestri, T. Ata Aydin, Melanie Wald-Fuhrmann

**Affiliations:** Max-Planck-Institute for Empirical Aesthetics, Frankfurt-am-Main, Germany

**Keywords:** Music cognition, Acoustic, Perceptual features, Cross-cultural, Categorization, Neuroscience, Psychology, Psychology

## Abstract

Making sense of our auditory environments involves forming meaningful perceptual categories. While ‘music’ is culturally diverse, Western listeners seem to consistently distinguish it from other sounds. In preregistered experiments, we examined the identification of sounds as music by presenting a large range of stimuli—musical excerpts from different geographical origins and a variety of other auditory materials—to a total of 735 online Western participants. Our findings demonstrate that sound evaluation is rather consistent, with a modest effect of listeners’ perspective, stimulus duration, and repetition on music categorization. Listeners tend to agree in their ratings and three distinct categories emerged: music, not-music, and ambiguous sounds. Within the music cluster, listeners’ evaluations of culturally diverse stimuli did not differ from the evaluations of genres more familiar to Western listeners. Although the three sound categories overlap in a low-level acoustic space, they were clearly separated by perceptual judgments—particularly the recognition of musical instruments, detection of melodies, and inference of intentionality. Such findings support the idea that ‘music’ is a distinct and stable perceptual construct encompassing a wide range of stimuli.

**Supplementary Information:**

The online version contains supplementary material available at 10.1038/s41598-026-54414-2.

## Introduction

What is music? Numerous music critics, theoreticians, and aestheticians have tackled this question since Antiquity^1,2^, and different academic disciplines have offered varying perspectives^3^. For instance, researchers have attempted to define it through objective properties, seeking either universal acoustic characteristics across cultures^4^ or features distinguishing music from other domains like speech^5–8^. Yet despite these efforts, no consensus has emerged, underscoring the need for alternative approaches to investigate what constitutes 'music. Rather than seeking a definitive ontological answer, we approach this question empirically: Does ‘music’ function as a coherent perceptual category, that is, do listeners reliably and consistently identify certain sounds as music despite surface diversity? On the one hand, historical musicology and ethnomusicology have both emphasized the great diversity of musical types, styles, and concepts^9^, which seems to render the existence of a homogeneous music category dubious. On the other hand, humans’ ability to group information into structured (and ultimately labelled and meaningful) categories is a common perceptual phenomenon that has been recurrently observed in multiple domains^10^. Categorization is usually demonstrated for objects, colors, or short events such as phonemes^11^ but it also occurs for more complex concepts such as moral judgments or knowledge^12^. Whatever the nature of the category, it is assumed that its members are consistently identified as being part of it by a (large) group of people.

The grounds on which listeners categorize sounds as music could be theorized through two contrasted lenses. An essentialist perspective posits that a category is defined by specific low-level acoustic features of the “object,” which reliably distinguish one category from the others, resulting in a strong acoustic-category mapping as it is the case for screams (i.e., strong role of roughness in scream/alarm perception^13,14^. By contrast, a constructivist approach holds that music emerges through shared exposure from an early age^15^, independently of strict acoustic correlates. This predicts a weak or absent acoustic-to-category mapping, as observed in emotional prosody^16^, singing preference^17^, and the speech-to-song illusion^18^. Instead, it would be associated with information such as high-level features (e.g., its function^19,20^, or perceptual features^17,21,22^. Hence, if music is a constructed category, diverse sounds should be judged as music (or not) consistently, with substantial agreement across listeners, even when stimulus properties and context vary widely.

Regardless of the theoretical perspective, the existence of a distinct music category implies that listeners categorize sounds consistently, that is, with limited variability across individuals and contexts. Among the potential sources of such variability, one can identify subjective, objective, and contextual factors. For instance, it has been shown that participants’ individual perspectives shape their judgments (e.g.,^23^ for moral judgment or^24^ for decision-making), supporting that participants/listeners’ perspective (i.e., their own view or others’ view) might shape the nature of sound categories. Another potential factor affecting sound categorization might be associated with the stimulus duration or amount of information available, and repetition. Indeed, studies in the auditory domain support the hypothesis that brief stimuli contain enough information for participants to recognize a musical style^25^, as well as to predict listener preference and familiarity of longer performances^26^. However, this may only apply to styles that are already familiar to listeners. Contrarily, unfamiliar musical styles might require stimuli of longer duration to be interpreted as being music or not music. Regarding the repetition effect or familiarity with the stimulus, previous work has shown high consistency of participants’ evaluations of the correctness of singing performances when they were heard twice^27^, but other studies also report changes in terms of recognition, familiarity, and liking over time^28^.

In four preregistered experiments, we tested whether music functions as a distinct perceptual category by examining the consistency of categorization across conditions. More specifically, we designed experiments testing the effect of listeners’ perspective, as well as the effect of stimulus duration and repetition, on sound categorization. We compiled a diverse set of 90 audio stimuli from music and sound online databases (description on https://github.com/pauline-lm/The_sound_of_music)  originating from different sound sources (humans, animals, nature, artifacts; but excluding vocalizations) and geographical locations in an effort to include potentially ambivalent stimuli. We included culturally varied excerpts to enhance generalization of findings to material potentially less familiar to Western listeners and to reduce the general Western bias in music research and psychology (see^29,30^ for discussion of this topic). Moreover, we investigated the stimulus characteristics that shape sound categorization. Specifically, we quantified (a) computationally extracted low-level acoustic features and (b) listeners’ ratings of theoretically grounded perceptual features (following Bruder’s et al. approach^17^. A robust acoustic-to-category mapping would support an essentialist view of music, whereas reliance on perceptual features would align with constructivist accounts—where music is defined by cultural consensus rather than low-level acoustic invariants.

## Results

### *Music* ratings are consistent

**Fig. 1:**
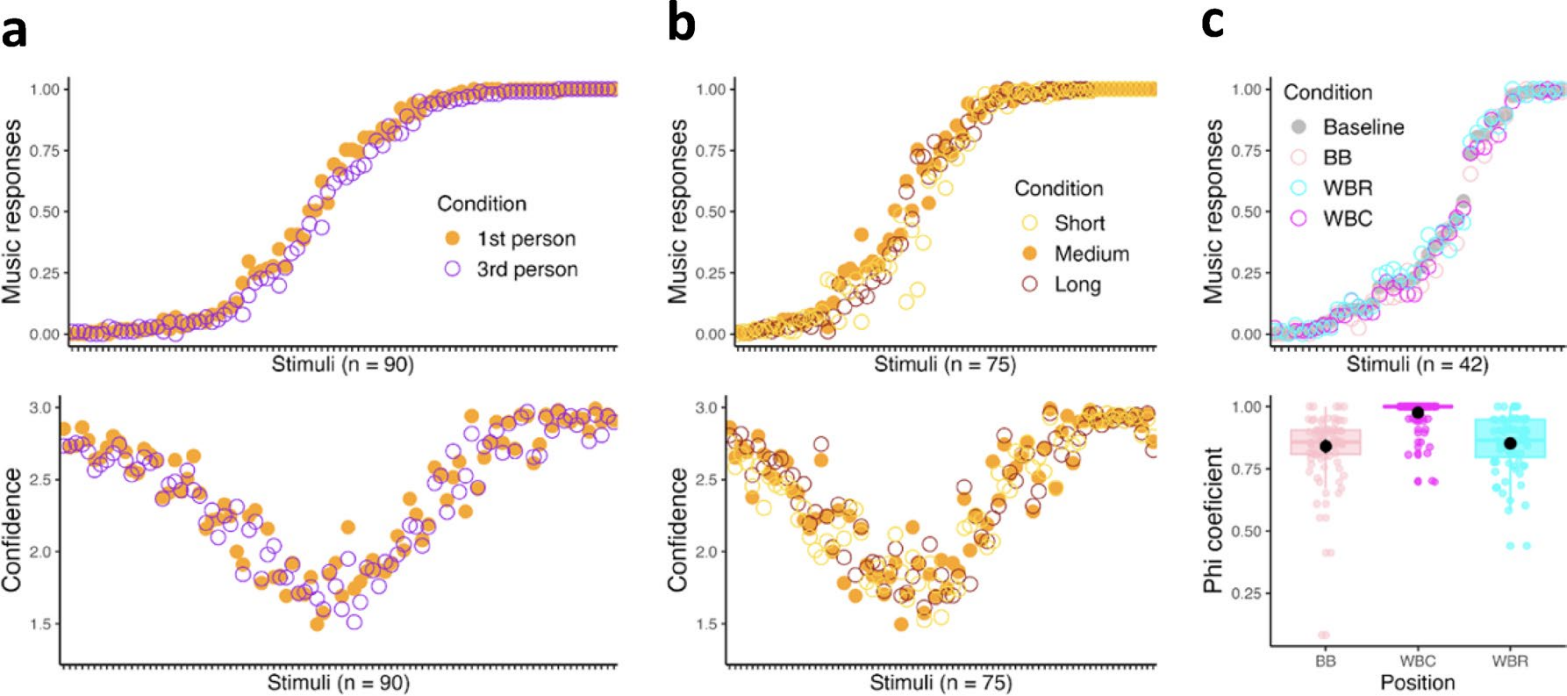
Results of experiments testing the effect of (**a**) listener’s perspective (Exp. 1), (**b**) stimulus duration (Exp. 2), and (**c**) repetition (Exp. 3; BB = Between Blocks; WBR = Within Block Random; WBC = Within Block Consecutive) on sound categorization. Top graphs: mean proportion of ‘music’ responses per stimulus (*n* = 90, 75, and 42, respectively, for each experiment) across conditions (colors), ranked by increasing mean for the reference condition (1st person / medium duration / baseline). Bottom graphs a and b: mean confidence ratings (0–3 Likert scale) per stimulus, following the same ranking. Bottom graph c: Phi coefficients estimating listeners’ consistency between first and second presentation across the three repetition conditions.

In three preregistered studies, we presented several carefully curated stimuli (n = 42 to 90) to Western online participants, asking them to classify each stimulus as either ‘Music’ or ‘Not music.’ We designed contrasting conditions in which participants took a 1st versus 3rd person perspective when evaluating the stimuli (Exp. 1), heard stimuli of different durations (Exp. 2), or heard each stimulus twice, with the interval between repetitions varying across conditions (Exp. 3). As is visible in Fig. 1a–c (top), the average ratings of each group of participants or condition (each group represented by a color) were similar, following a sigmoid shape, with plateaux at the extremes of the scale, representing stimuli judged as either highly musical or not musical. Note that the plateaux contained stimuli that were rated with particularly high confidence, as illustrated in Fig. 1a, c (bottom). On the not-music side of the spectrum we found sounds from machines (such as a coffee grinder, a sewing machine or a typewriter), but also from water (a river, rain), while stimuli combining a clear melody with instrumental accompaniment, harmonic progressions, and a distinct rhythm received the highest ‘music’ ratings.

Listener’s perspective, stimulus duration, and repetition each had a small but significant effect on sound categorization. Including ‘condition’ as a fixed effect improved model fit in all three experiments (Exp. 1: χ^2^(1) = 5.59, *p* = .018; Exp. 2: χ^2^(2) = 12.70, *p* = .002; Exp. 3: χ^2^(2) = 3.94, *p* = .047). The comprehensive models suggest a bias towards ‘music’ responses when listeners’ answers reflect their own perspective, as opposed to what they predict a third person’s judgment would be (Fig. 1a, 1st person > 3rd person, *z* = 2.383, *p* = .017), when stimuli have a *medium* duration of 5 seconds long, compared to *longer (10 seconds)* and *shorter (2 seconds)* ones (Fig. 1b, medium > long, *z* = −2.172, *p* = .030, medium > short, *z* = −3.575, *p* < .001, long = short, *p* > .05), and when the stimuli are presented a second time (Fig. 1c, z = 2.012, *p* = .044), with fewer changes in answers when the second presentation follows directly the first one (Within Blocks Consecutive: WBC) than when it comes later in the same block (Within Blocks Random: WBR) or in another block (Between Blocks: BB). Phi coefficients differed significantly across repetition conditions (*F*(2, 237) = 40.46, *p* < .001; Tukey HSD: WBC < WBR = BB), and correlations between Phi coefficients across conditions ranged from .84 to .97. However, all effects were particularly small. Across all three repetition conditions, listeners rarely revised their categorization upon re-hearing a stimulus, suggesting that music categorization is stable regardless of the temporal distance between presentations. In addition, a substantial proportion of the variability in ratings was explained by stimulus-level random effects (82.7%, 81.6%, and 73.9% for WBC, WBR, and BB, respectively), reflecting the deliberately wide range of sounds selected. In other words, the role of conditions was minor given the overall results, supporting the consistency of music categorization responses.

Finally, the explorative examination of the 33 stimuli that were rated by all the participants of experiments 1–3 (*n* = 637) confirmed that the proportion of participants categorizing a stimulus as music or not was similar across conditions. In fact, the ratings of the 33 stimuli across conditions were highly correlated (mean correlation coefficient of .98, all *ps* < .001, see correlation matrix of the Supplementary Information SI1 for pairwise coefficients), supporting once again the high consistency in the music categorization responses. Interestingly, listeners seem to be relatively confident in their ratings (all above 1.5 on a Likert scale from 0 to 3) whatever the answer and the condition (i.e., adding the variable ‘Condition’ in the models predicting confidence did not enhance their quality). High confidence was particularly visible for the stimuli on the two plateaux at the extremes of the music scale (see U-shaped graphs representing participants’ confidence in their judgments in Fig. 1a and b, bottom).

### Salience of *music*, *not-music*, and *ambiguous* categories

**Fig. 2:**
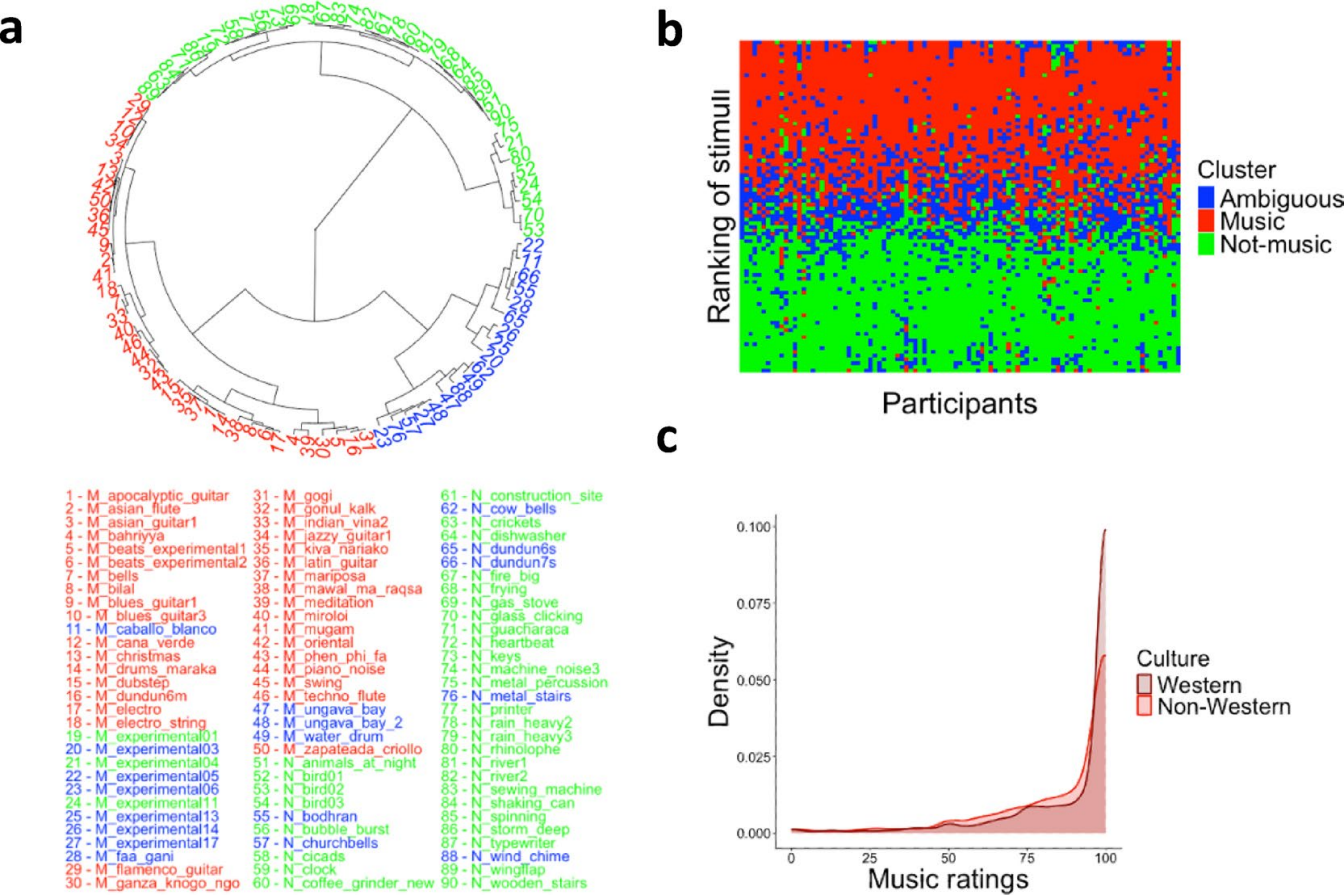
Clusters of stimuli identified by k-means analysis in Exp. 4. (**a**) Dendrogram representing grouping similarity across the 90 stimuli; branch height reflects dissimilarity. Colors indicate cluster membership: music (red, *n* = 36), not-music (green, *n* = 36), and ambiguous (blue, *n* = 18). (**b**) Consistency of cluster membership across participants (*n* = 98 columns); the y-axis represents each participant’s stimulus ranking, colored by cluster. (**c**) Distribution of music ratings within the music cluster, separately for Western and non-Western excerpts.

To substantiate further the sigmoidal curves from Exp. 1–3, together with the U-shaped confidence ratings from Exp. 1–2 (Fig. 1), we carried out a fourth experiment in which a new sample of 98 Western participants rated each stimulus on a slider, ranging from ‘not music’ to ‘music.’ Using a slider allowed for more granularity in listeners’ responses. Prior to Exp. 4, a control experiment confirmed that continuous (as in Exp. 4) and binary response formats (as in Exp. 1–3) converge at the group level (see SI2 for methods, results, and discussion of the control experiment). A k-means cluster analysis, performed on participants’ average answers, revealed a three-group solution that can be described as *music*, *not-music*, and *ambiguous* categories (Fig. 2A). The music cluster (*n* = 36) had a mean music rating of 87.5 (*SD* = 7.77), the not-music cluster (*n* = 36) of 12.8 (*SD* = 7.33), and the ambiguous cluster (n = 18) of 52.4 (*SD* = 9.59).

The music and not-music categories each contain 36 stimuli. Interestingly, the ambiguous category (*n* = 18 stimuli) is composed of stimuli either representing types of music that vastly differ from Western standard forms (such as experimental music or indigenous music from Mexico and Canada), metallic sounds such as bells or wind chimes, and Nigerian speech surrogate drum sounds. These stimuli correspond to those found on the slopes of the sigmoid curves in Fig. 1 (top panels), that is, sounds for which listeners showed the greatest variability in their categorization responses across Exp. 1–3, neither consistently rated as music nor as not-music. Note that the three clusters are consistent across individuals though there are also isolated colored dots for some participants, as illustrated by the pattern of colors of the 90 stimuli ranked in Fig. 2b. This result, in line with the Krippendorff’s alpha of 0.68 (computed on the raw ratings), supports a moderate agreement between participants. Interestingly, as seen in Fig. 2c, the distributions of music ratings for both groups of stimuli (i.e., Western vs. non-Western) overlap within the music cluster (*n* = 36), in which half of the excerpts originated from non-Western geographic locations. A linear mixed effects model confirmed that Western listeners’ music ratings are not affected by the origin of the excerpt, *β* = 3.51, *SE* = 2.56, *t*(34) = 1.37, *p* = .179, supporting that within the music category, cultural origin of the stimuli do not influence Western listeners’ ratings.

### Perceptual (rather than acoustic) features predict sound categories

To explore what shapes Western participants’ music category, we examined how low-level acoustic characteristics and perceptual features predicted sound categorization. We chose to combine these two types of features for methodological reasons. Low-level acoustic features were extracted computationally, following the long-standing tradition in music information retrieval (MIR) of characterising audio signals through a broad set of low-level descriptors. When it comes to higher-level perceptual attributes, computational extraction remains arguable, since the correspondence between the extrated estimates and human perceptual judgments is generally weak, as demonstrated in singing research^17^ and music perception more broadly^31,32^. We therefore relied on listener ratings to capture higher-level features, rather than on computational estimates. Except for the extraction itself, the same procedure was followed for both acoustic and perceptual feature sets: the number of features was reduced to two principal components (see Methods for details, see SI3 for feature loadings), which were then entered as fixed effects in two separate cumulative link models predicting the three stimulus clusters.

**Fig. 3:**
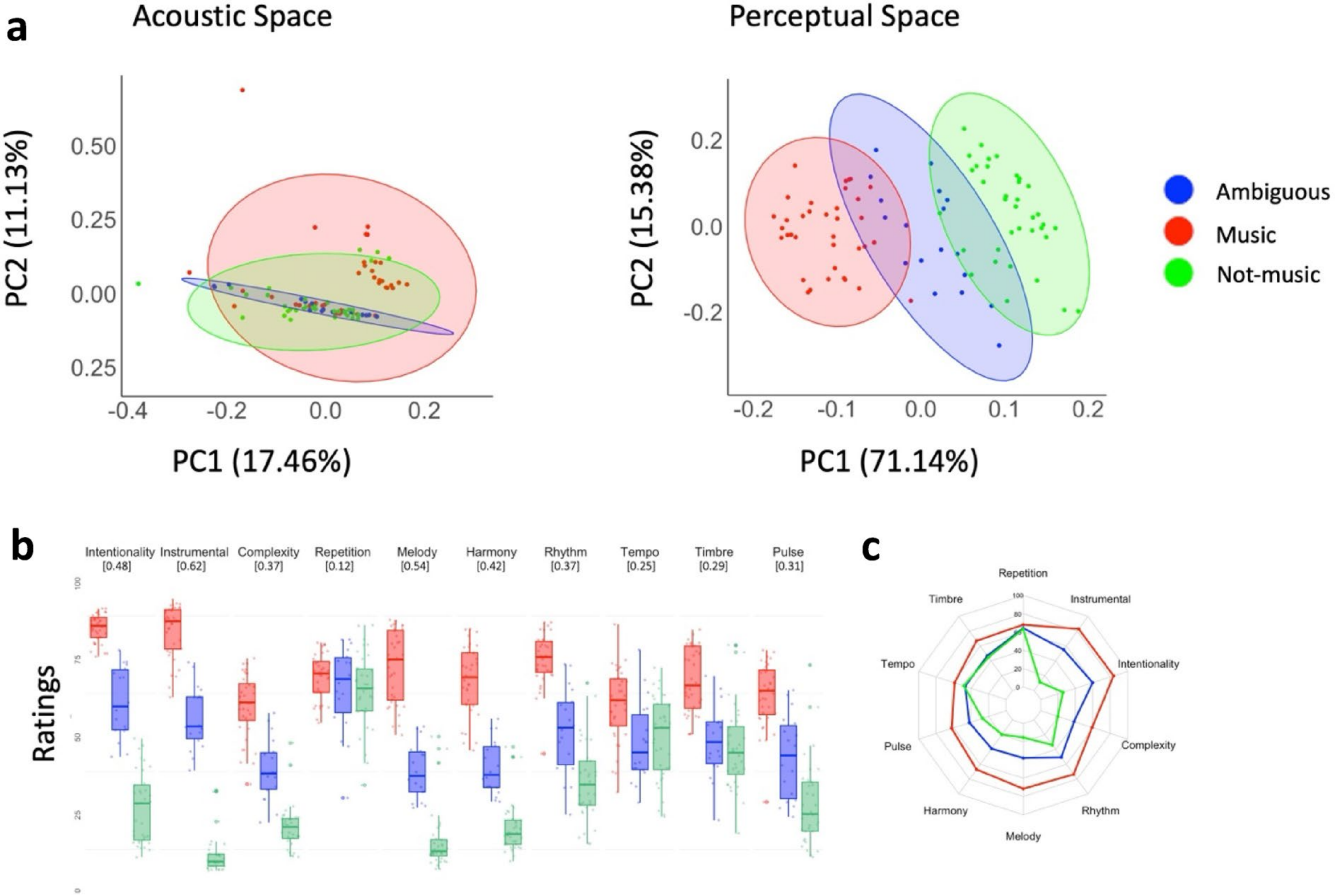
Feature spaces and perceptual profiles of sound clusters. (**a**) Position of stimuli in the two-dimensional acoustic (left) and perceptual (right) spaces; colors indicate cluster membership (music = red, not-music = green, ambiguous = blue). (**b**) Boxplots of stimulus ratings for each perceptual feature by cluster; Krippendorff’s alpha values (inter-rater agreement) are displayed below each label. (**c**) Radar chart summarising mean feature ratings by cluster.

Both the acoustic and perceptual models outperformed the null model that only included random effects (acoustic: χ^2^(2) = 22.34, *p* < .001; perceptual: χ^2^(2) = 167.84, *p* < .001). The perceptual model showed a better fit than the acoustic one, explaining substantially more variance in music ratings (Nagelkerke pseudo R^2^ = 96.17% vs. 25.02%; see Table SI4 for full model comparison). In the acoustic model, PC1 (β = 0.079, *SE* = 0.03) and PC2 (β = 0.25, SE = 0.08) were both significant predictors (*p* < .05). Both dimensions were also significant in the perceptual model (PC1: β = 6.27, *SE* = 2.33; PC2: β = 2.76, *SE* = 1.09; both *p* < .05). Note that alternative feature extraction methods yielded highly similar results (see SI4 for descriptions of alternative approaches and their respective model outputs). As illustrated in Fig. 3a, the three stimulus clusters overlap considerably in the low-level acoustic space with music stimuli spanning its entirety, whereas the perceptual space cleanly separates music, not-music, and ambiguous stimuli, with ambiguous stimuli occupying the region between the other two. In line with Bruder and colleagues^17^, these findings suggest that music categorization relies more heavily on higher-level perceptual judgments than on low-level acoustic properties.

The exploration of individual perceptual features in a comprehensive perceptual model (see Methods) allows us to identify the factors grounding the music ratings. Figures 3B–C suggest that the greatest differences among the three categories are associated with the higher-level features (e.g., perceived intentionality of sounds; attribution of the sound source as a musical instruments). The model predicting the music ratings from the ratings of individual perceptual features also outperformed the null model (*χ*^2^(10) = 1101.9, *p* < .001), confirmed this interpretation by supporting the role of intentionality (*β* = 0.077, *SE* = 0.010), instrumental content (i.e., presence of instrument) (*β* = 0.152, *SE* = 0.011), repetition (*β* = −0.030, *SE* = 0.010), melody (*β* = 0.129, *SE* = 0.012), and rhythm (*β* = 0.047, SE = 0.011) in predicting the variance of music ratings (all *p* < .05). The perceptual features of the models accounted for 15.74% of the variance, and the global model reached 65.74% with addition of the variance explained by the random intercepts for participants and stimuli.

## Discussion

Taking an empirical stance on the perennial question ‘What is music?’ and focusing on Western listeners as a first step, we observed that listeners form a consistent and stable music category. Manipulations of perspective, stimulus properties, and context (Exp. 1–3) produced only slight flexibility, supporting the view that music is a robust perceptual category. The music category included excerpts from varied geographical locations. Even though we only focused on Western listeners, this finding is in line with cross-cultural studies showing that listeners use similar cues whatever the origin of the material (e.g., when categorizing vocal stimuli as speech or song, see^33^. Future studies are essential to determine when the stability we observed in the categorization of sounds as music emerges and whether it extends beyond Western populations, particularly given well-documented variations in musical concepts across cultures^4,9^.

The *music* category was driven predominantly by perceptual features rather than low-level acoustic characteristics. Our conclusions contrast with those of studies distinguishing song (as a form of music) from speech^8,34^ but are in line with neuroscientific findings suggesting that music-selective cortical responses cannot be fully accounted for by standard acoustic features^35^. The weak acoustic-to-category mapping observed here is in line with results from emotional prosody research showing a complex mapping between the speech signal and paralinguistic meaning^16^. The current results demonstrate the nuanced relationship between low-level acoustic features and the concept of music, distinguishing music from other non-verbal auditory material that shows a stronger acoustic-meaning mapping^13^. Importantly, the primacy of perceptual features aligns with findings that musical preferences are predicted by listeners’ interpretation of sound, rather than by low-level signal properties^17^. While aesthetic appreciation and music categorization are two different tasks, both point to a strong role of top-down influences. This role is further supported by the nature of the strongest predictors in our model (e.g., intentionality) and by the fact that stimuli from non-Western cultures and less familiar genres (e.g., experimental music) with less clear intentionality or instrumental content tended to fall outside the music category, even though the stimuli within it are highly varied (see SI5).

The ambiguous category deserves particular attention, as it marks the boundary of the music category. Excerpts in this cluster did not share a common perceptual profile; each possessed only a subset of the ten investigated features. For instance, experimental and indigenous music (e.g., excerpts from Mexico and Canada) lack clearly recognizable instruments or conventional melodic structure. Metallic sounds such as bells and wind chimes exhibit pitch and periodicity but carry no perceived intentionality. In the case of Nigerian speech surrogate drumming, timbral cues suggest a musical instrument, yet the rhythmic patterns follow the irregular prosodic contours of speech rather than the periodic structures typically expected of music, resulting in categorization uncertainty. What these stimuli share is an incomplete perceptual profile. They do not activate some of the higher-level cues that drive music categorization, such as intentionality, instrumental content, or a clear melodic structure. The ambiguous category thus represents an in-between state, where excerpts neither fully possess nor lack the cues investigated (Fig. 3b), possibly reflecting listeners’ ambivalence when evaluating perceptual features. Note that we observed relatively low inter-rater agreement for perceptual feature ratings, which may reflect diversity in how participants relied on different cues. Future studies could explore additional individual factors (e.g., social, cultural, or cognitive characteristics) that might explain contrasting strategies in the use of perceptual features. The role of higher-level features observed here (e.g., instrumental content) is also aligned with ethnomusicological perspectives. The relevance of intentionality resonates with Blacking’s foundational characterization of music as “humanly organized sound,” foregrounding human agency over acoustic structure^36^. The role of intentionality raises critical questions about the role of human agency in music production, especially given the increasing use of artificial intelligence (AI) tools in creative domains^37^.

Although our work was conducted thoughtfully (and transparently, through preregistration), several methodological constraints warrant discussion. For instance, we can legitimately question the completeness of the acoustic analysis, including our reliance on default extraction settings, which may not be optimal for such a diverse stimulus set. We made an initial attempt to address this by comparing several feature extraction approaches and tools (see SI4), and the convergent results across these alternatives suggest that our conclusions are not tied to a specific computational pipeline. A related limitation concerns the correspondence between computationally extracted features and human perceptual judgments. Although we deliberately separated low-level acoustic features (extracted computationally) from higher-level perceptual features (measured through listener ratings), future work could further clarify how these two levels relate to one another^31,32^ for instance, by identifying which acoustic properties underlie listeners’ judgments of intentionality, instrumental content, or melodic structure. Future implementations could incorporate more dynamic acoustic and perceptual features, refining computational models to better predict music categorization (for such an approach in the visual domain, see^38^.

Another limitation is that our stimulus set consisted of relatively short, isolated sound excerpts presented without visual or social context—a design that was necessary to ensure experimental control but that departs considerably from how music categorization typically unfolds in everyday life. In ecological settings, listeners rarely encounter sounds in isolation and top-down influences can powerfully modulate category judgments—a sound that might be classified as noise in one context could readily be accepted as music in another (e.g., Cage’s conceptual redefinition of environmental sounds as music, or the well-documented context effects in auditory scene analysis^39^. Since listeners usually categorize sounds without specific labels being presented, using free sorting tasks might be more ecologically valid (e.g.,^40,41^ though participants might still have a specific context in mind that could vary over time. Whether the stability and nature of the perceptual category of music observed here remains in more naturalistic settings is thus an open question.

In conclusion, our findings challenge essentialist approaches that seek to define music through a fixed set of low-level acoustic criteria. Instead, the data are more consistent with a constructivist framework in which “music” is a perceptual category grounded in listeners’ higher-level interpretations of sound—particularly the recognition of musical instruments, the detection of melodic structure, and the inference of intentionality. This empirical picture converges with long-standing positions in the humanities. The category structure we observe, that is, no single defining feature, but overlapping clusters of perceptual cues that different stimuli share to varying degrees, closely mirrors Wittgenstein’s concept of family resemblance, in which category membership is sustained not by necessary and sufficient conditions but by a network of partial similarities^42^. Relatedly, Weitz argued that “art”—and by extension “music”—functions as an open concept, inherently resistant to closed definitions because new instances can extend its boundaries^43^. The ambiguous stimuli in our data, which include experimental music and unfamiliar indigenous traditions, illustrate precisely this openness; repeating the same experiment at a later point in time might further illuminate changes in perception of sounds as music. Together, these convergences suggest that the empirical study of categorization and the humanistic analysis of music as a cultural concept need not remain separate enterprises. The present data offer evidence that “music” is not a natural kind defined by acoustic invariants, but a culturally shaped, listener-dependent construct—stable enough to generate broad agreement within a community of listeners, yet flexible enough to accommodate the remarkable diversity of the world’s musical practices.

## Methods

### Experimental design

To address our research questions (Table 1), we employed within- and between-subjects designs tailored to each hypothesis. Preregistered analyses included modeling specific effects (Exp. 1–3) and, for Exp. 4, identifying stimulus clusters as well as examining acoustic/perceptual predictors of sound categories.

**Table 1:** Design of the experiments testing the effects of listeners’ perspective (Exp. 1), stimuli duration (Exp. 2) and repetition (Exp. 3), on the identification of stimuli as music or not, and investigating the role of acoustic and perceptual features on music ratings (Exp. 4).

Exp.	Question	Pre-registration	Condition	Participants	Stimuli: number (duration)
1	Listeners’ perspective	osf.io/sb24q	First- versus third-person perspective	200	90 (5s)
2	Stimulus duration	osf.io/azkcp	Short Medium/first-person perspective Long	298 (including 101 from Exp. 1)	75 (2s, 5s, 10s)
3	Stimulus repetition	osf.io/re7dg	Repetition Between Blocks (BB) Within Block Random (WBR) Within Block Consecutive (WBC)	240	42 (5s)
4	Role of perceptual and acoustic features	osf.io/hqj4s	N/A	98	90 (5s)
SI	Effect of response format on music evaluation	N/A	Binary (first-person perspective of Exp. 1) versus Slider	95	90 (5s)
SI	Variability of the music samples	N/A	Pairwise comparisons of similarity	64	36 (5s)

In addition, two control experiments were proposed to compare response formats and investigate the variability of the music samples (presented in detail in SI2 and SI5). We report and discuss simplified versions of the preregistered analyses.

### Open practices statement

Preregistrations can be found on the OSF (Table 1). Analyses that deviated from the preregistration are reported in the text. Data collection took place between September 2022 and July 2024, after the individual pre-registration of each experiment. Data and analysis code can be found in the following repository: https://github.com/pauline-lm/The_sound_of_music. The experimental procedure was ethically approved by the Ethics Council of the Max Planck Society. All participants provided informed consent prior to participation, and all methods were performed in accordance with the relevant guidelines and regulations.

### Participants

Participants’ information was collected at the end of sessions of all experiments and is reported in Table 2. Countries of online recruitment were: Austria, Switzerland, Germany, UK, France, Luxembourg, Italy, Denmark, Finland, Norway, Ireland, Spain, Liechtenstein, Poland, Netherlands, Belgium, Czech Republic, Portugal, and Sweden. There was no restriction regarding biographical characteristics (e.g., age, gender, socio-demographic background, musical training). The experiments were, however, only open to individuals showing a validation rate above 95% in their previous participation to online studies through the Prolific platform^44^ (www.prolific.com). Note that participation was not validated in cases of: incompletion of the task and questionnaires; presence of self-reported hearing impairments; or failure at the attention/quality check (see procedure section for details). Upon validation, participants were compensated at the rate of 9 UK pounds/hour.

Due to a lack of previous empirical studies of this topic with the proposed design, the sample size was not grounded on a power analysis but relied on our experience with online behavioral studies using the Prolific platform for the first experiment. The sample size of the new groups was decided to match this sample size and allow for comparable variance across conditions.

**Table 2:** Characteristics of all the participants (*n* = 637) who completed the online experiments.

Exp.	Number	Age Mean (min-max, SD)	Self-reported gender	Music sophistication Mean (min-max, SD)	Vividness abilities Mean (min-max, SD)
1	200	27.84 (19–65, 8.36)	Female: 73 Male: 121 Other: 6	70.78 (18–122, *SD* = 18.10)	4.82 (1–7, *SD* = 0.98)
2	298 (including 101 from Exp. 1)	29.03 (19–67, 9.43)	Female: 98 Male: 194 Other: 4 No answer: 2	71.57 (18–126, *SD* = 18.55)	4.81 (1–7, *SD* = 1.03)
3	240	27.29 (19–62, 8.17)	Female: 104 Male: 129 Other: 6 No answer: 1	68.16 (22–112, *SD* = 18.02)	4.66 (1–7, *SD* = 1.10)
4	98	34.98 (18–74, *SD* = 11.32)	Female: 30 Male: 68	71.46 (29–114, *SD* = 19.78)	4.79 (1–6.93, *SD* = 1.02)

The music sophistication index was estimated with a subset (*n* = 18 items) of the Gold-MSI^45^ and the vividness abilities were estimated with the BAIS_V scale of the questionnaire designed by Halpern^46^. The participants of the different experiments do not significantly differ in terms of self-reported music sophistication and vividness abilities (both ANOVAs on the scores: p > 0.05).

### Material

Ninety audio files were curated to sample broadly from the space of everyday auditory experience. Selection followed two orthogonal principles: First, stimuli were chosen to maximize cultural and geographical diversity, drawing on everyday sounds and musical traditions from multiple world regions (including East and South-East Asia, the Middle East, Sub-Saharan Africa, Latin America, Arctic/Indigenous communities, and Western Europe and North America). Second, stimuli were selected to systematically represent distinct sound source categories—human-produced sounds, animal sounds, natural environmental sounds, material sounds (e.g., metal, wood), and sounds from artifacts and machines. Note that human vocalizations were excluded because of their particularly salient auditory signal with specialized neural processing mechanisms (e.g.,^47^), which could bias listeners’ evaluation of such stimuli. This two-dimensional sampling strategy was designed to ensure that the resulting stimulus set would probe the boundaries of the music category across both culturally familiar and unfamiliar musical traditions and across a wide range of non-musical auditory materials.

Audio tracks were collected from Smithsonian Folkways Recordings (https://folkways.si.edu/), Wiki-Commons (https://commons.wikimedia.org/), Pixabay (https://pixabay.com), Free Music Archive (https://freemusicarchive.org/), PacDV (https://www.soundeffectsplus.com), and previous studies^48–50^. Excerpts of 5s duration (exact reference and timing can be found following the link: https://github.com/pauline-lm/The_sound_of_music) as well as 2s and 10s duration for Exp. 2, were extracted with Audacity (version 3.0.2) from the original files. To avoid bias towards music ratings, selection of the shorter and longer duration stimuli disregarded the potential musical phrases contained in the audio material. Stimuli were then normalized (adjustment of volume with Peak normalization to 0.0 dBFS), and pre-processed (fade in of 0.2 and fade out of 0.6 s) with To Audio Convertor software (version 1.0.16). For Exp. 2 and 3, a subset of stimuli was selected based on the findings from Exp. 1 to shorten the task while keeping variability in responses. In addition to the set of 90 stimuli, a barking sound was included in the context of the quality check of the participants (see Procedure section).

Based on the origins of stimuli as stipulated in the description of the datasets, we further divided stimuli included in the “music cluster” (see Exp. 4) in two groups: Western and non-Western. The non-Western excerpts originated from East and South-Eastern Asia, Middle East, North Africa, Sub-Saharan Africa, Latin America and the Caribbean, and an Arctic/Indigenous region (Nunatsiavut); Western excerpts included pieces originating from Portugal, USA, and Greece. Please note that we use the terms “Western” and “non-Western” to operationalize excerpts in two groups, serving as a proxy (though not empirically tested) of European listeners’ familiarity with the stimuli.

### Procedure

All experiments were designed in Labvanced^51^ (www.labvanced.com). Participants were presented with the description of the study and granted access to the main task after providing informed consent. We used an attention check in this main task to validate each individual’s participation. For this purpose, barking sounds (about 10% of the total number of trials) were randomly presented and, on these occasions, participants were asked to select a specific answer (“Music” box for Exp. 1–3 or “Music” side as well as right end of all scales for Exp. 4). Note that we emphasized this instruction by saying that our objective was to make sure that participants would be attentive throughout the task. Also, participants had the option to click on the “Back to Instructions” button during the task itself if they wished to re-read them. Participation was validated when more than 80% of trials were correctly completed (or 80% of answers on the 1/5 right side of the slider for Exp. 4). For all experiments, once the participants had answered (progress to the next page required a response), they could press “Next” and the next page (with the next audio file) was presented. The task was divided into two blocks with self-paced breaks in between.

#### Specificity of Exp. 1

Participants were randomly assigned to the *1st person* (*n* = 101) or *3rd person* (*n* = 99) conditions. For the *1st person* condition, after hearing each of the 90 audio files, played randomly, participants were asked to answer the question “What do you think this is?“; for the *3rd person* condition, they were asked to answer the question “What would most people think this is?” In each case, participants selected the response “Music” or “Not music.” For each trial, participants were asked to rate their confidence in their answer on a four-point ordinal scale from 0 (not at all) to 3 (absolutely).

#### Specificity of Exp. 2

Participants were randomly assigned to the *short* (*n* = 99, stimuli of 2 s long) or *long* (*n* = 98, stimuli of 10 s long) conditions. The *medium* condition consisted of the *1st person* condition of Exp. 1, that is, participants (*n* = 101) evaluated 5 s long audio files. For all three conditions, participants were asked to answer the question “What do you think this is?” after listening to each of the randomly presented 75 audio files by selecting the response “Music” or “Not music,” and to estimate their confidence level on the 0–3 scale.

#### Specificity of Exp. 3

Participants were randomly assigned to the *Between-Blocks* (BB, *n* = 81, repetition in the second block) or *Within-Block-Random* (WBR, *n* = 79, stimuli were presented twice within the same block but the interval between the two presentations was randomized) or *Within-Block-Consecutive* (WBC, *n* = 80, the second presentation occurred immediately after the first one). For all conditions, after listening to each of the selected 42 audio files, participants were asked to answer the question “What do you think this is?” and could select the response “Music” or “Not music”. Unlike in Experiments 1 and 2, the question about confidence was not proposed.

#### Specificity of Exp. 4

Participants were asked to answer “What do you think this is?” using a slider presented on the screen (0 = Not-music, 100 = Music). Our change of the response format was motivated by the wish to focus on individual differences and was decided after an additional experiment allowing direct comparison of the output of the forced-choice answer format (from Exp. 1, *1st person* condition) with a new sample of 98 online participants, who were instructed in exactly the same way except that the answer would be continuous on a slider. Prior to conducting Exp. 4, we ensured that both binary responses (as in Exp. 1–3) and slider (as in Exp. 4) converge to similar results at the group level in a control experiment (see Supplementary Material SI2 for detail on the methods and results). The main finding is that the answer format had no significant effect on how participants evaluated the stimuli. We can thus confidently interpret the findings of Exp. 4 (Fig. 2A) as replicating the data collected in Exp. 1–3.

For this experiment, a second task was proposed in which participants heard each stimulus a second time and answered several questions about perceptual attributes of the stimuli on individual sliders with anchors at both ends. A list of descriptors that could potentially describe music stimuli was curated. Inspired by^17^, we tried to include features that could have phenomenal correspondence to lay listeners. In addition, we incorporated complexity because of its relevance in predicting preference for auditory stimuli^52^. The definitions of the selected features are as follows:

- **Pulse**: A pulse means that a sound sequence has a beat in principle you can tap along to *(No pulse–Pulse)*.
- **Complexity**: Complexity refers to a sound that is made of a small/large number and variety of events. *(Simple–Complex)*
- **Melody**: A melody is a meaningful sequence of tones of various length. *(No melody–Melody)*
- **Instrumental**: The sounds were played on (a) musical instrument(s). *(Not instrumental–Instrumental)*
- **Timbre**: Timbre refers to the perceived quality of the sound, such as its “color” or “brightness”. *(Dark–Bright)*
- **Tempo**: Tempo refers to the speed of the sound sequence. *(Slow–Fast)*
- **Intentionality**: A sound sequence is intentional if it was produced on purpose. *(Not intentional–Intentional)*
- **Harmony**: Harmony refers to the presence of multiple layers/tones at the same time that belong together and complement each other. *(No harmony–Harmony)*
- **Rhythm**: A rhythm consists of a temporal pattern of sound events repeated multiple times. *(No rhythm–Rhythm)*
- **Repetition**: Repetition refers to looped events. Note that due to an implementation error, this definition was not presented in the instructions but participants reported having understood the definition at the end of the experiment. *(Not repetitive–Repetitive)*

For all experiments, following the main task, the Bucknell Auditory Imagery Scale (BAIS-V) questionnaire was used to quantify auditory imagery abilities^46^. The 14 BAIS-V questions required participants to rate how clearly they could imagine a particular auditory image (e.g., a trumpet beginning to play “Happy Birthday”) from 1 to 7, with 1 indicating that no image was present at all, 4 being fairly vivid and 7 being as vivid as the actual sound. Responses on the subscales were averaged across items. Participants were then asked to answer questions from a short version of the Gold-MSI (*n* = 18 items) in order to quantify their musicality^45^. For Exp. 4, participants were also asked to rate how well they understood the definitions presented (on 7-point scales). Finally, for all experiments, a general questionnaire was proposed to all participants to capture basic biographical information (e.g., age, gender, country of residence) and their point of view regarding the task itself.

### Statistical analyses for Exp. 1–3

We used a modeling approach to test the effect of the condition (i.e., *1st person* vs. *3rd person* in Exp. 1; *short* vs. *medium* vs. *long* in Exp. 2; *first* vs. *second presentation* in Exp. 3) on participants’ responses (i.e., “Music” or “Not music”). A mixed-effects logistic regression (glmer from lme4 package) was applied to predict the response from the condition, with random intercepts for *participants* and *stimuli items*. Note that the reported model differs from the preregistered one, which included the “a priori group” of stimuli as fixed effect in the tentative equation. The output of the proposed model is consistent with what was expected but, for clarity, we report here a simpler version of the model that actually tests our hypothesis more specifically.

To assess the contribution of the fixed effect under study, we compared the full models, which includes the condition as a fixed effect along with the random effects, to a null model, which includes only the random effects. To understand the proportion of variance in the ratings attributable to the random effects in the model, we calculated the Intraclass Correlation Coefficient (ICC) using the performance package in R^53^. The confidence ratings (in Exp. 1 and 2) were also examined with a similar approach but with the consideration that the confidence rating is an ordinal variable, not graduated linearly but with responses scaling in one direction and with steps that are not necessarily equal (following the recommendation of^54^ and thus using the ordinal package for Cumulative Link Mixed Models (CLMMs^55^).

### Statistical analyses for Exp. 4

#### Cluster analysis

A k-means cluster analysis (kmeans function from the *stats* package, Euclidian distance methods) was proposed to identify clusters of stimuli based on the mean music ratings of stimuli across participants. The number of clusters was defined by visual inspection of the scree plot (elbow method) and examination of the within-cluster sum of squares between successive numbers of clusters.

#### Acoustic model

Acoustic feature extraction was performed using Essentia’s *Freesoundextractor* (v2.1 beta2)^56^ (https://essentia.upf.edu/). The initial extraction resulted in 248 features. Essentia generated pre-computed descriptors across multiple feature groups: low-level spectral features, rhythm, sound effects, and tonal features. We used default extraction settings throughout. We acknowledge that the acoustic diversity of our stimulus set might benefit from fine-tuned extraction parameters; however, systematically optimising settings across such a varied corpus would quickly become intractable given the number of features involved. To reduce the dimensionality of the feature set, we performed a Principal Component Analysis (PCA) and used the first 2 principal components (PCs). The first two PCs accounted for a cumulative explained variance of 28.59% (see SI3 for details). These dimensions were then used as predictors of a music category (3 = music, 2 = ambiguous, 1 = not-music) in a cumulative link model (*ordinal* package, clm(category ~ PC1 + PC2)). This model, referred to as the “Acoustic model”, was compared to a null model (i.e., with random effects only) to estimate the contribution of acoustic dimensions to stimulus cluster identification.

#### Perceptual model

Regarding the acoustic parameters, the number of perceptual features was reduced with a PCA and the resulting dimensions were used as predictors of the music ratings with the same method as the acoustic model (*ordinal* package; syntax: category ~ PC1 + PC2). The first two dimensions of the PCA based on the selected features, accounted for a cumulative variance explained of 86.52%. The precise number of features and their loadings on each dimension can be found in SI3. The model, referred to as the “Perceptual model,” was compared to a null model, which includes only the random effect, in order to estimate the contribution of perceptual dimensions in the identification of stimuli as being music or not.

#### Exploratory analyses for Exp. 4

In addition to the aforementioned analyses, we describe the agreement measures for music ratings and the ratings of perceptual features (Krippendorff’s alpha^57^); and the generalized linear mixed model (*lme4* package) to investigate the effect of stimulus origin on listeners’ ratings (ratings ~ origin + (1 | participants) + (1 | stimuli)).

Finally, previous work has revealed large degrees of variability in participants’ use of perceptual features^17^. Therefore, for transparency, we also decided to report a complementary exhaustive perceptual model using the music ratings as a dependent variable, all perceptual scales as fixed effects, and the participants as well as stimuli as random effects.

## Supplementary Information

Below is the link to the electronic supplementary material.

Supplementary Material 1

## Data Availability

All data and code used in the analyses, as well as extensive information about the study materials, are publicly available on https://github.com/pauline-lm/The_sound_of_music..
