# Supplementary material for: Music is a distinct perceptual category with subjective grounds
Source: Sci Rep. 2026 May 27;16:16414. doi: 10.1038/s41598-026-54414-2 (PMC13216321; doi:10.1038/s41598-026-54414-2)
Supplement: Supplementary file 1 — Supplementary Material 1 [file 41598_2026_54414_MOESM1_ESM.docx]

Supplementary Materials for

Music is a distinct perceptual category with subjective grounds

**This PDF file includes supporting information about:**

SI1. Analyses of Exp. 1-3

SI2. Control Experiment: Binary versus slider response format

SI3. Principal Component Analysis (PCA) applied to the perceptual and acoustic characteristics of the stimuli (Exp. 4)

SI4. Fit indices and explained variance for perceptual and acoustic models predicting stimulus clusters (Exp. 4)

SI5. Control Experiment: Variability of the music samples

References SI2-SI5

**SI1. Analyses of Exp. 1-3**

A subset of 33 stimuli were common across the three first experiments and evaluated by a total of 637 participants. Figure SI1.1 supports the stability of stimuli ratings across conditions and the very limited role of participants' characteristics on the bias towards music rating (in line with the results from Exp. 4 reported in the main text).


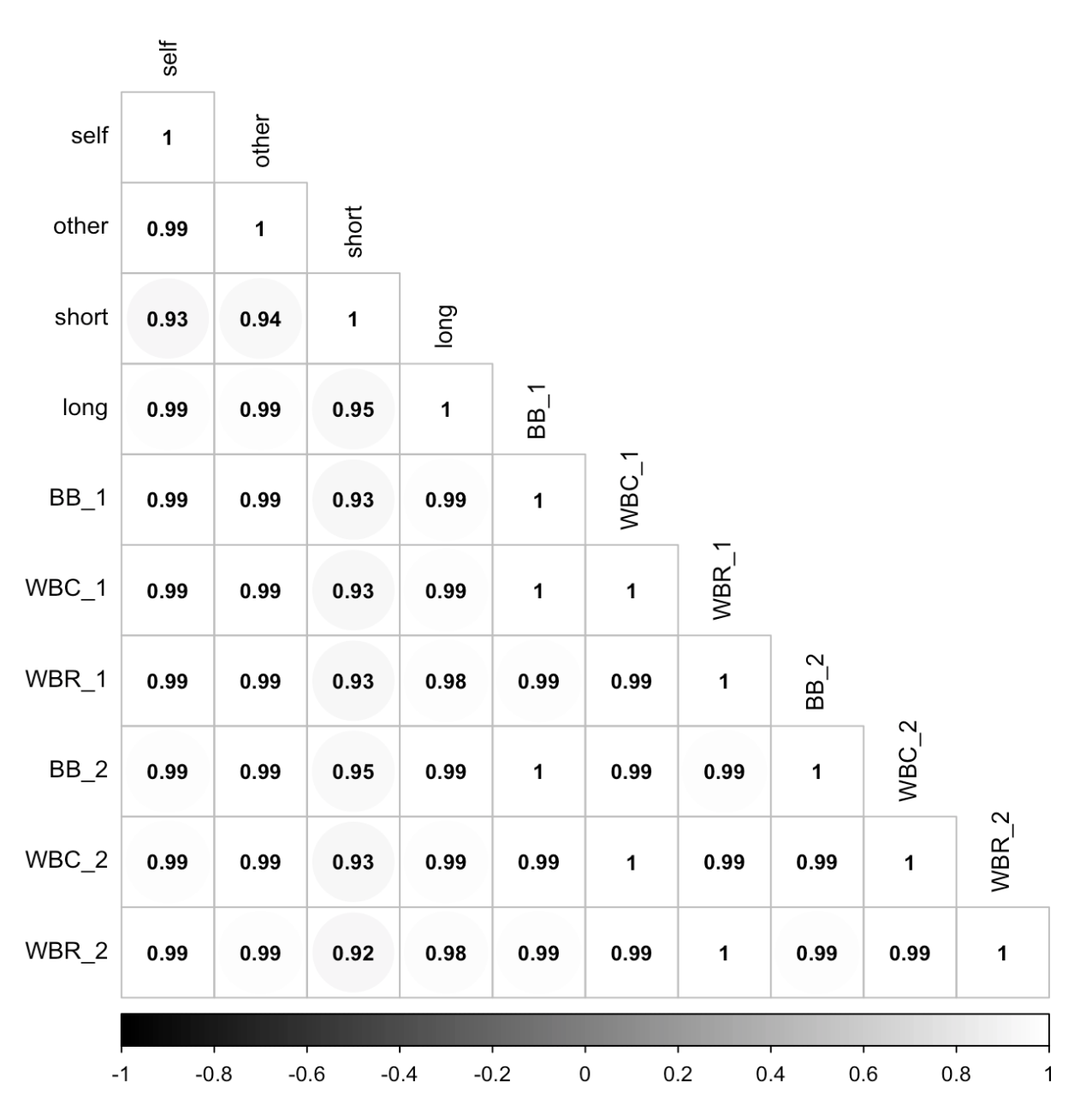


**Figure SI1.1.** Consistency of stimuli evaluation (n = 33) across experiments (Exp. 1-3) illustrated with a matrix of Pearson correlation coefficients between the different conditions, ranging from .92 to 1. Grey scale representing the magnitude of the correlation coefficient; All *ps* < .001. For Exp. 3, the second presentation (noted as _2) occurred in the second block (Between Blocks, BB), in the same block, right after the first presentation (Within Blocks Consecutive, WBC), or in the same block but at random position (Within Blocks Random, WBR).

**SI2. Control Experiment: Binary *versus* slider response format.**

The experimental design of Exp. 1-3 used a binary forced-choice format. Concretely, participants were asked to listen to each stimulus, read the question: "How does it sound to you?" and selected the "music" or "not music" answer. Through these experiments, we varied the instruction (Exp. 1), the duration of the material (Exp. 2), and the mode of presentation (Exp. 3), but kept the answer format constant. For the analyses, the binary data (0: not-music; 1: music) were used as dependent variables in the linear mixed effect models proposed to examine the effect of the conditions on the participants' evaluation of the stimuli. In addition, we computed the proportion and mean of "music" answers across participants to illustrate the sigmoid-like shape of the data (Figure 1). However, using binary data hides the potentially graded nature of listeners' judgments.

In Exp. 4, we aimed at capturing the gradience in the ratings by using a slider ranging from "not music at all" to "very much music" as a response format. Whereas direct comparisons of response format in music perception research are rare [*1*], literature in adjacent domains highlight advantages and disadvantages of both. Sliders provide informative output but participants with lower educational backgrounds seem to have a higher break-off rate and seem to need more time to complete the task [*2-4*]. On the other hand, forced choice tasks are straightforward but might affect the output, by inflating the number of participants who have opinions on a matter [*5*] or leading to more "agreement" with the direction of the question [*4*].

In this control experiment, we test whether the response format affects the categorization of **sounds** by comparing the answers provided with a binary forced choice task (Exp. 1, *1st person* condition, referred to as "binary group") with the data of a new group of participants evaluating the exact same stimuli, reading the same question (i.e., "What do you think this is?"), but answering on a pseudo continuous 100-point scale that visually resembles a slider (referred to as "slider group").

Methods

**Participants.** Ninety-five online participants (*M*_age_ = 30.34 *SD*_age_ = 9.40) were recruited following the same criteria as for Exp. 1-3.

**Material.** The exact same dataset of 90 5s-long stimuli was used (doi: 10.17617/3.I9BJQ1).

**Procedure.** As for the binary group, participants of the slider group listened to the stimulus and were asked "What do you think this is?". However, they had to answer on a slider from 0 (not music) to 100 (music) instead of a binary forced-choice. All other settings were kept the same: consent, instruction, order of the stimuli (randomized for every participant), presentation in two blocks, self-paced pause, and questionnaires at the end. Also, the attention check was comparable: Every time participants heard a barking sound *(n* = 10 in total), they were asked to put the cursor to 100 (instead of selecting "Music" in Exp. 1-3). Only participants with a mean score above 80 are included in the analyses.

**Analysis.**To directly compare the two conditions (i.e., forced-choice versus slider), we proposed a linear mixed effect model with the music answers as a dependent variable, the condition as fixed effect, and participants and stimuli as random effects. Note that for this analysis, we used the original output from the forced-choice condition and inferred the binary answer in the slider condition (not music if < 50, music if ≥ 50) as music answers. This transformation allows us to use the "binomial" family of the lme4 package [*6*], in the following syntax: music answer ~ Condition + (1|Participant) + (1|Stimuli), family = binomial. This main analysis was completed with the computation of Spearman correlation coefficients at the group level between the scaled mean ratings of both conditions as well as between the participants of the slider condition. Indeed, the binary format leads to one data point per stimulus (and thus only allows to examine the range of music answers at the group level) but the slider allows further investigation of potential individual differences in stimuli ratings.

Results and discussion

The evaluation of stimuli from not-music to music (in the slider condition) seems similar to the scaled mean answers computed by averaging the group's binary answers (here the output of Exp. 1 *1st person* condition is plotted under the name "forced-choice", Figure SI2a). In line with the output of Exp. 1-3, some stimuli are clearly considered as not-music, some are clearly considered as music, and several stimuli are in the middle range of the scale.


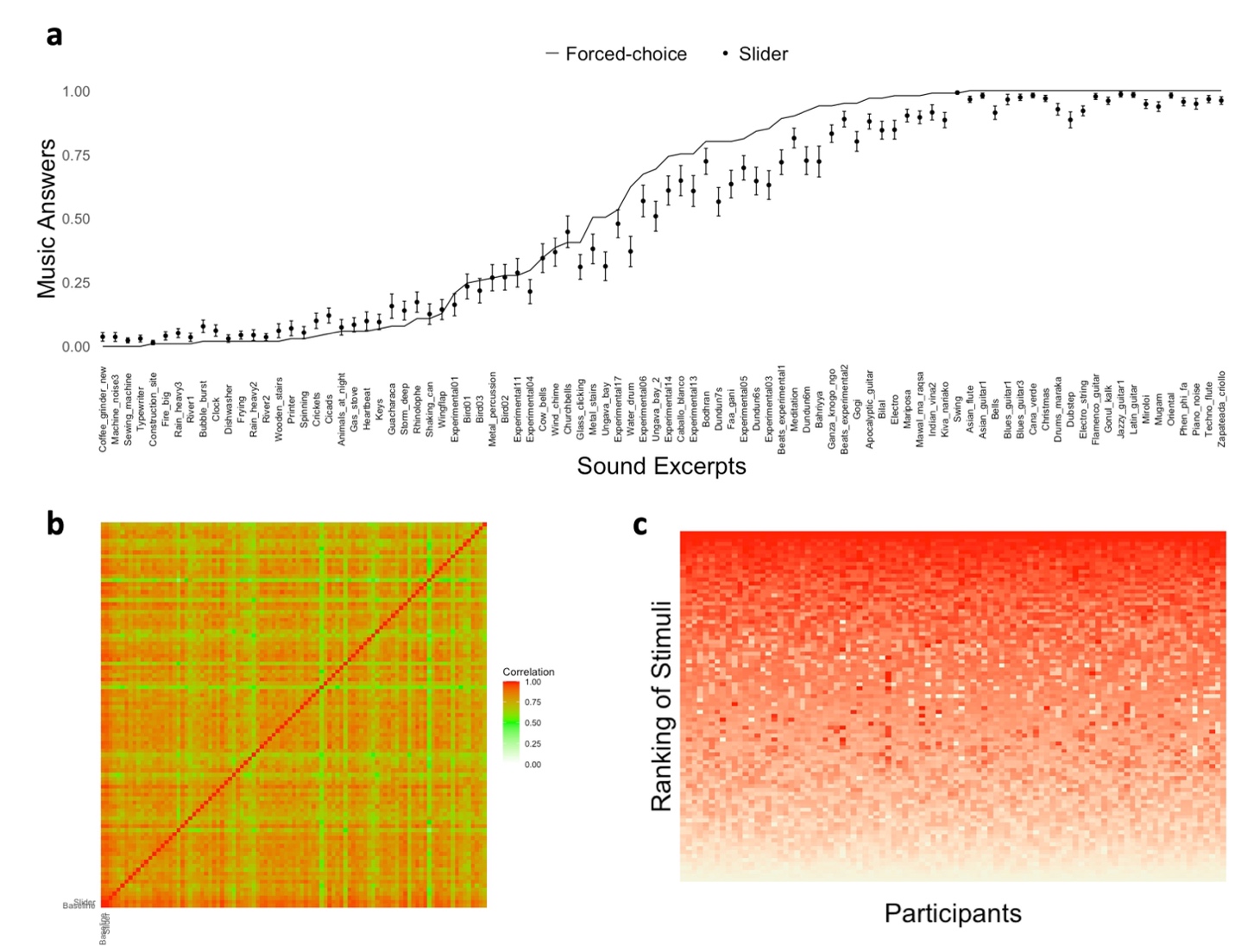


**Figure SI2.1.** Illustration of the results of the control experiment designed to examine the effect of response format on music evaluation. **a)** Scaled mean music rating for each of the 90 stimuli in the two conditions (i.e., forced-choice versus slider), ranked according to the mean scaled rating across conditions. For the slider condition, dots correspond to the mean rating across participants and error bars stand for the 95% Confidence Interval. **b)** Correlation matrix of the group (i.e., forced-choice binary versus slider) and individual rankings (*n* = 95, slider only) of the 90 stimuli under study. **c)** Rankings of the rating of each stimulus (y axis), with the first column representing the ranking of mean music ratings across participants (*n* = 95), and all the other columns representing each participant. The darker represent higher music ratings (ranging from 0 to 100).

The mixed effect model revealed an effect of condition on music ratings, with slightly more "music" answers in the binary forced-choice condition (*z* = 2.53, *p* = .01, *β* = .38, *SE* = .15) compared to the slider condition. Though the question is not whether participants agree or not with a statement, the answer "music" could be seen as the positive option. In this case, the result is in line with the effect observed in other fields [*4*], with inflation of the "agreement with the question" option in the case of binary choices. However, the distribution of the scaled mean ratings in both conditions (i.e., forced-choice versus slider) are similar (Figure SI2.1) and mean ratings of the stimuli between conditions are highly correlated (*r*(88) = .99, *p* < .0001), see the red cell in the left/bottom of Figure SI2.1b). Altogether, these results support that the effect of format exists, but remains limited when looking at the group level.

In addition to providing information about the mean music answer for each stimulus, the slider format allows to gain insights about potential individual differences not visible when using a binary choice format (though it is still possible to examine the bias towards music answers for some participants). Figure SI2.1c represents the ratings of the stimuli per participant (x axis) colored from light (low rating) to dark (high music rating) red. Individual differences are actually minimal, as visible with the homogeneous shade across columns (i.e., participants) and highlighted by the low "participant" variance as random effect in the model (conditional R^2^ = 0.82, marginal R^2^ = 0.001, participant's variance = 3.4%, stimuli variance = 84.6%). Concretely, the variance in the data stems from the between-stimulus difference, which was expected since we carefully constructed a diverse stimulus set.

Altogether, this control experiment highlights that the two response formats (binary forced-choice versus slider) provide convergent results at the group level. Changing the original response format to a slider thus has a limited effect on the evaluation of stimuli as music or not, while enhancing granularity in participants’ responses.

**SI3. Principal Component Analysis (PCA) applied to the perceptual and acoustic characteristics of the stimuli (Exp. 4)**

A principal component analysis was carried out for both the perceptual (n = 10) and the acoustic features (n = 248). This was done to reduce the number of predictors, and the obtained dimensions were later used in linear mixed effects models to predict the music ratings provided by the participants of Exp. 4.

PCA for the perceptual characteristics

A PCA was carried out on the perceptual features to reduce dimensionality. The first two dimensions cumulatively explained 86.52% of variance in the data (Figure SI3, left), and were used for further modeling. All features loaded on the first dimension except for tempo and repetition, which loaded on the second principal component (Table SI3).


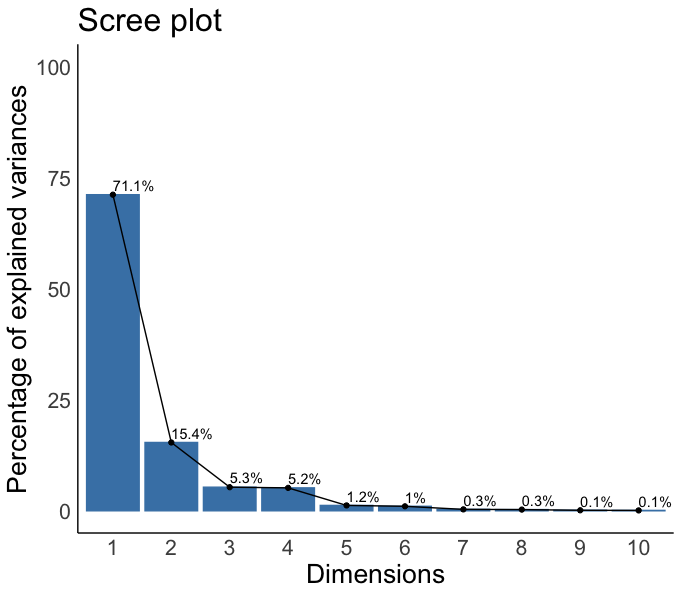

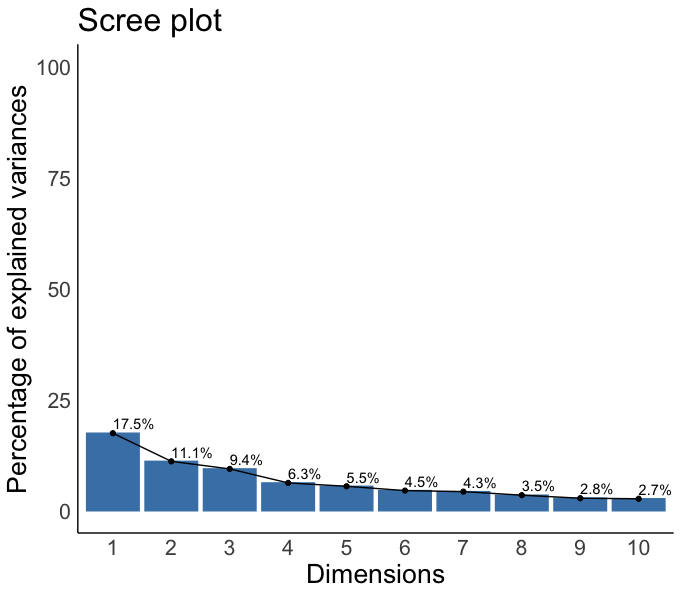


**Figure SI3.** Scree plot showing the proportion of variance explained by each principal component, based on the ratings of the 10 perceptual features (left) or on the 248 extracted acoustic features (right).

*Table SI3. Loadings of the mean ratings of perceptual features on the first two principal components.*

| **Variable** | **PC1** | **PC2** |
| --- | --- | --- |
| Intentionality | **0.36** | -0.12 |
| Harmony | **0.36** | -0.17 |
| Rhythm | **0.36** | 0.13 |
| Melody | **0.36** | -0.18 |
| Instrumental | **0.35** | -0.17 |
| Pulse | **0.34** | 0.17 |
| Complexity | **0.33** | -0.21 |
| Timbre | **0.29** | 0.003 |
| Tempo | 0.18 | **0.55** |
| Repetition | 0.13 | **0.71** |

PCA for the acoustic characteristics

Dimensions 1 (17.5%) and 2 (11.1%) together explained 28.6% of the total variance. To remain consistent with the number of dimensions selected for the perceptual model, we retained only these two dimensions. Note that including additional ones would not substantially increase the variance explained (Figure SI3.1). Feature loadings on PC1 and PC2 consisted of timbral descriptors (GFCCs, MFCCs) and spectral-shape descriptors (flatness, entropy, flux, spread, kurtosis, rolloff, energy, low-frequency energy, Bark-band spread). Note that the majority of these features captured the mean and variance of a descriptor across time frames rather than its static value. PC1 and PC2 therefore reflect the temporal dynamics of timbral and spectral properties, that is, how these characteristics change from frame to frame, rather than their average magnitude.

**SI4. Fit indices and explained variance for perceptual and acoustic models predicting stimulus clusters (Exp. 4)**

*Table SI4. Model fit indices for the acoustic and perceptual cumulative link models predicting sound category. Higher R² values and lower AIC/BIC indicate better model fit.*

| **Fit index** | **Perceptual model** | **Acoustic model** | **Alternative model 1** | **Alternative model 2** | **Alternative model 3** |
| --- | --- | --- | --- | --- | --- |
| Nagelkerke pseudo R² | 96.17% | 25.02% | 33.27% | 25.05% | 17.24% |
| McFadden pseudo R² | 88.39% | 11.77% | 16.39% | 11.78% | 7.79% |
| Cox and Snell pseudo R² | 84.51% | 21.98% | 29.24% | 22.01% | 15.15% |
| AIC | 30.04 | 175.54 | 166.76 | 175.51 | 183.10 |
| BIC | 40.04 | 185.54 | 176.76 | 185.51 | 193.10 |
| Variance explained by the first 2 PCs (in %) | 86.5 | 28.6 | 22.3 | 26.6 | 26.6 |

In addition to the perceptual and acoustic models described in the main text (and in the first two columns of Table SI4), we applied models using alternative sets of acoustic features, extracted with either Essentia [7] (Alternative models 1 and 2) or MIRtoolbox [8] (Alternative model 3). For Alternative model 1, Essentia's MusicExtractor resulted in 916 features across three groups (i.e., low-level spectral, rhythm, and tonal) summarized as the mean and standard deviation across the default analysis frames of MusicExtractor (frame size: 2048 samples/~46 ms for low-level features and 4096 samples/~93 ms for tonal features, at a sampling rate of 44,100 Hz). For Alternative model 2, we selected only the low-level features from the MusicExtractor output, that is, 708 descriptors. For Alternative model 3, 279 features were extracted using MIRtoolbox (v1.8.2) in MATLAB R2025b with a frame size of 50 ms for most descriptors, and longer frames of 100–200 ms for chroma and novelty-based features. Six summary statistics were computed across frames (mean, standard deviation, minimum, maximum, median, and linear slope) for 15 spectral descriptors: spectral centroid, RMS energy, brightness, flatness, zero-crossing rate, pitch, roughness, spectral rolloff, spread, skewness, kurtosis, regularity, entropy, low energy, and spectral flux. For vector-valued features, mean and standard deviation were computed per coefficient or band: 13 MFCCs and their delta coefficients, 12 chroma bins, and 10 mel-band energies. Spectral flux was additionally computed across 10 sub-bands. Global descriptors were also extracted: tempo, pulse clarity, key clarity, mode, harmonic change detection function, spectral novelty, fluctuation, and attack time.

For each alternative model, we followed the same procedure as for the original models: dimensionality reduction with PCA, retention of the first two components, and inclusion in a cumulative link model predicting stimulus clusters.

**SI5. Control Experiment: Variability of the music samples**

In Exp. 1-4, we curated a set of stimuli that reflected various types of sounds from different geographical locations. Exp. 4 supports that the 90 stimuli of the set can be divided into three clusters: *music*, *not-music*, and *ambiguous* categories. Here, we focus on the “music” cluster and examine the perceived heterogeneity of the stimuli. In other words, this experiment was carried out to quantify the variability of the 36 stimuli of the music category. European online participants were asked to evaluate the similarity of pairs of stimuli on a slider, from 0 to 100. Our assumption was that high similarity between groups of stimuli (and dissimilarities with others) would allow us to identify sub-groups of music (that would be worth further investigating) whereas low similarity would reflect our effort to select contrasted material.

Methods

**Participants.** Sixty-four online participants (*M*_age_ =  33.36, *SD*_age_ = 11.36) were recruited using Prolific ([9], www.prolific.co) applying the same criteria used in Exp. 1-4. All participants were compensated with 9£ per hour if they provided complete data and succeeded (more than 80% correct answers) the attention check.

**Material.** The material used in this experiment consisted of the 36 stimuli of the music cluster (as a result of the cluster analysis in Exp 4). The sound excerpts were pseudo-randomly concatenated in pairs with a 1.7 seconds long interval in between [10]. After guaranteeing that no stimulus was paired with itself and there were no pairs in reverse order (e.g., S1-S2, but not S2-S1), the total number of stimuli was 630.

**Procedure.** The experimental task was implemented using Labvanced [11]. Since the total number of pairs (n = 630) was too high to be evaluated by each participant, the stimuli were pseudo-randomly distributed to 6 different conditions, with each condition having 105 trials and 10 attention checks, which consisted of a barking sound that participants had to rate as “Completely similar.” In each condition, participants were asked to rate how similar they thought the presented stimulus pair was on a slider from 0 (*not similar at all*) to 100 (*completely similar*). Since similarity could be interpreted differently, the following instruction was provided to aid participants’ evaluation: “For example, if you think about fruits, one might rate oranges to be more similar to lemons, but less similar to apples. Or peaches are more similar to pears compared to tomatoes.” A break was proposed after the 50^th^ trial. After completing the experimental task, the rest of the experiment was identical to Exp. 1-3.

**Analysis.** We examined the distribution of similarity ratings and assessed the percentage of variance explained by each potential factor (participants, stimuli) with a linear mixed model: *similarity ~ 1 + (1 | stimuli) + (1 | participants).* Note that we also calculated the Krippendorff’s alpha for each condition (i.e., group of participants) to estimate interrater agreement in similarity ratings.

Results and discussion

Figure **SI5**.1 illustrates that pairs of stimuli were rated as rather dissimilar (*M* = 32.85, *SD* = 23.14, on a slider from 0 to 100). In fact, only 12.22 % of the 630 pairs had a mean rating above 50. In line with the literature suggesting limited interrater agreement in music similarity ratings [12], the mean Krippendorff’s alpha for all six conditions was .11 (ranging from to .04 to .16). Additionally, the linear mixed model confirmed participants’ different approaches while evaluating the stimuli and showed that the 30% of variance in similarity ratings was explained by participants and 16% of variance was coming from the stimuli.


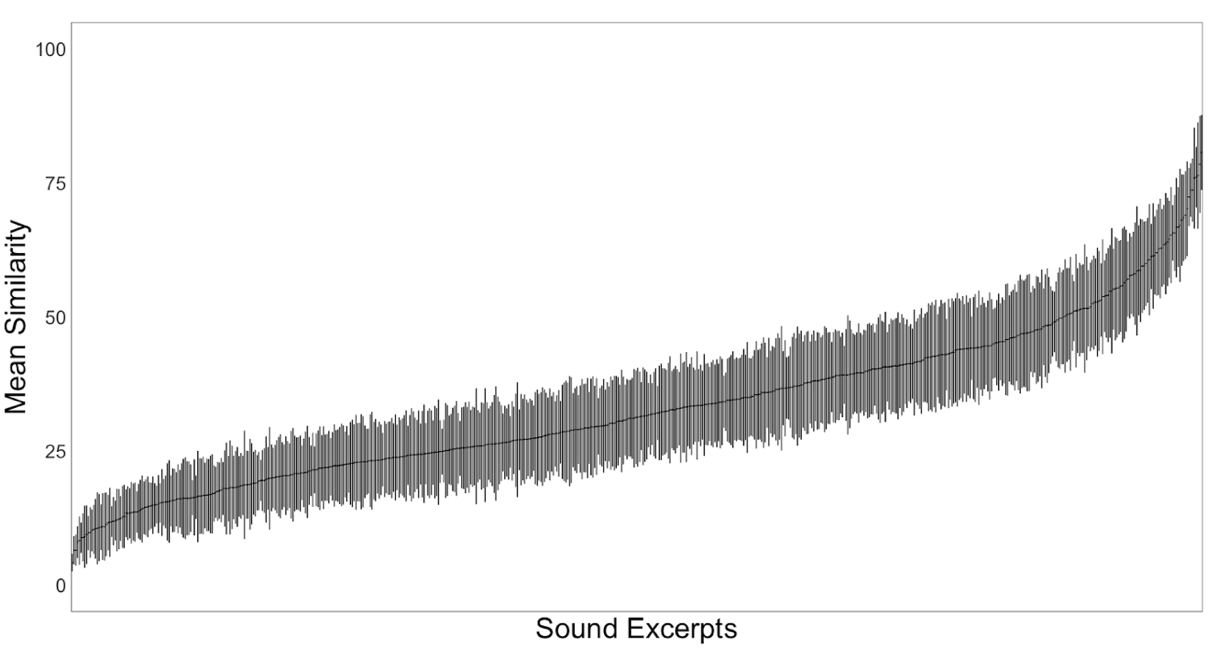


**Figure SI5.1.** Mean (and SD) similarity ratings for each of the 630 pairs presented, ranked from lowest to highest. Most pairs have a mean similarity below 50.


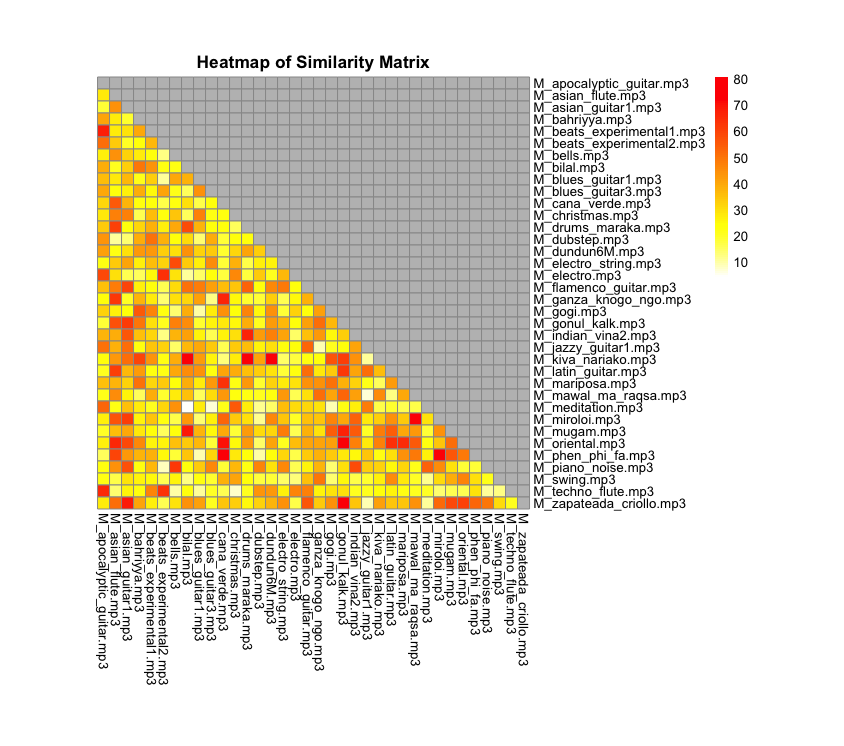


**Figure SI5.2.** Similarity matrix of all 36 music stimuli. Yellow shades display dissimilarity and red ones suggest that the stimuli of the pair are similar to each other.

The pairs with the highest similarity ratings (e.g., phen phi fa and cana verde, gonuk kalk and zapateada criollo) suggest that similarity ratings might be mainly driven by instrumental timbre (such as in pairs of plucked string instruments, drums or shawms), way of playing (such as tremolando), mode (as in pairs set in non-Western modes) or general character (such as dance-like rhythms in a moderate to fast tempo). Altogether, the descriptive statistics and similarity matrix confirm that the stimuli interpreted as “music” by listeners (see results of Exp.4) represented a diverse selection.

**References SI2-SI5**

[1] Brittin, R. V. Listeners’ preference for music of other cultures: comparing response modes. *J. Res. Music Educ.* 44, 328–340 (1996).

[2] Funke, F. A web experiment showing negative effects of slider scales compared to visual analogue scales and radio button scales. *Soc. Sci. Comput. Rev.* 34, 244–254 (2016).

[3] Funke, F., Reips, U.-D. & Thomas, R. K. Sliders for the smart: type of rating scale on the web interacts with educational level. *Soc. Sci. Comput. Rev.* 29, 221–231 (2011).

[4] Rivera-Garrido, N., Ramos-Sosa, M. P., Accerenzi, M. & Brañas-Garza, P. Continuous and binary sets of responses differ in the field. *Sci. Rep.* 12, 14376 (2022).

[5] Friedman, H. H. & Amoo, T. Rating the rating scales. *J. Mark. Manag.* 9(3), 114–123 (1999).

[6] Bates, D., Mächler, M., Bolker, B. & Walker, S. Fitting linear mixed-effects models using lme4. *J. Stat. Softw.* 67, 1–48 (2015).

[7] Bogdanov, D., Wack, N., Gómez, E., Gulati, S., Herrera, P., Mayor, O., ... & Serra, X. Essentia: an open-source library for sound and music analysis. In Proceedings of the 21st ACM international conference on Multimedia (pp. 855-858). (2013).

[8] Lartillot, O., Toiviainen, P., & Eerola, T. A matlab toolbox for music information retrieval. In Data Analysis, Machine Learning and Applications: Proceedings of the 31st Annual Conference of the Gesellschaft für Klassifikation eV, Albert-Ludwigs-Universität Freiburg, March 7–9, 2007 (pp. 261-268). Berlin, Heidelberg: Springer Berlin Heidelberg. (2008).

[9] Prolific. Quickly find participants you can trust. [https://www.prolific.com](https://www.prolific.com/) (2014).

[10] Muralikrishnan, R. Awk and shell scripts using ffmpeg for concatenating two sound files with a silence in between.

[11] Finger, H., Goeke, C., Diekamp, D., Standvoß, K. & König, P. LabVanced: a unified JavaScript framework for online studies. In *Proc. 2017 International Conference on Computational Social Science (IC2S2)* (2017).

[12] Flexer, A. & Grill, T. The problem of limited inter-rater agreement in modelling music similarity. *J. New Music Res.* 45, 239–251 (2016).
